# Supplementary material for: Rapid genome editing by CRISPR-Cas9-POLD3 fusion
Source: eLife. 2021 Dec 13;10:e75415. doi: 10.7554/eLife.75415 (PMC8747517; doi:10.7554/eLife.75415)
Supplement: Supplementary file 3. [file elife-75415-supp3.docx]

**Supplementary File 3:**Clustering of the hit-fusion’s protein interactions**.** Helicase activity pathways highlighted in orange color.

| **Category** | **Term** | **Count** | **%** | **P value** | **Genes** |
| --- | --- | --- | --- | --- | --- |
| GOTERM_MF_FAT | GO:0003729~mRNA binding | 14 | 12.173913 | 5.84E-10 | P15880, P84103, P16989, P22626, P27797, Q99729, P11940, Q92945, Q14103, O00425, P23396, P62854, P40429, P38159 |
| GOTERM_MF_FAT | GO:0003697~single-stranded DNA binding | 8 | 6.95652174 | 1.01E-05 | P33993, P33991, P10809, P16989, P22626, P61978, Q14566, P15531 |
| GOTERM_MF_FAT | GO:0003678~DNA helicase activity | 6 | 5.2173913 | 5.62E-05 | P33993, P33991, P49736, Q9Y230, Q9Y265, Q14566 |
| GOTERM_MF_FAT | GO:0004003~ATP-dependent DNA helicase activity | 5 | 4.34782609 | 1.70E-04 | P33993, P33991, Q9Y230, Q9Y265, Q14566 |
| GOTERM_MF_FAT | GO:0003677~DNA binding | 34 | 29.5652174 | 6.40E-04 | Q16531, Q9H0D6, P16989, P27797, Q9Y230, P23246, P09874, Q8IUE6, P78527, P0C0S5, P22392, P06899, Q92945, Q9UQ80, P49736, Q00839, P61978, P23396, Q14566, P84090, Q01105, P22626, Q8ND56, P06748, Q99729, Q16777, P15531, P33993, P33991, P16403, P10809, P78347, Q14103, P38159 |
| GOTERM_MF_FAT | GO:0008026~ATP-dependent helicase activity | 6 | 5.2173913 | 0.00106725 | P33993, P33991, Q9Y230, Q9Y265, Q92841, Q14566 |
| GOTERM_MF_FAT | GO:0004386~helicase activity | 7 | 6.08695652 | 0.00114469 | P33993, P33991, P49736, Q9Y230, Q9Y265, Q92841, Q14566 |
| GOTERM_MF_FAT | GO:0008094~DNA-dependent ATPase activity | 5 | 4.34782609 | 0.00300335 | P33993, P33991, Q9Y230, Q9Y265, Q14566 |
| GOTERM_MF_FAT | GO:0042826~histone deacetylase binding | 5 | 4.34782609 | 0.00681963 | P62258, Q14103, P31946, P09874, P23246 |
| GOTERM_MF_FAT | GO:0043139~5'-3' DNA helicase activity | 2 | 1.73913043 | 0.05743824 | Q9Y230, Q9Y265 |
| GOTERM_MF_FAT | GO:0003688~DNA replication origin binding | 2 | 1.73913043 | 0.07812389 | P10809, P49736 |
| GOTERM_MF_FAT | GO:1990837~sequence-specific double-stranded DNA binding | 10 | 8.69565217 | 0.08674837 | P0C0S5, Q9H0D6, P10809, P16989, P49736, Q9Y230, Q14103, P61978, P23246, P15531 |
