# Supplementary material for: Rapid genome editing by CRISPR-Cas9-POLD3 fusion
Source: eLife. 2021 Dec 13;10:e75415. doi: 10.7554/eLife.75415 (PMC8747517; doi:10.7554/eLife.75415)
Supplement: Supplementary file 5. [file elife-75415-supp5.docx]

**Supplementary file 5:**

CRISPR gRNA sequences of guides that target endogenous loci:

| **Targeted gene** | **Abbreviation** | **Location (Hg19)** | **sgRNA sequence** | **Guide binding strand** | **Gene orientation** |
| --- | --- | --- | --- | --- | --- |
| CCAT1 colon cancer associated transcript 1 | Enh 4-1 | chr8:128226403-128226422 | GTAGAATGTCAACTTCATGA | - | REV |
| Transcriptional repressor CTCF | CTCF1 | chr8:128746377-128746396 | TACTTTCGCAAACCTGAACG | + | N/A |
| Ring finger protein 2 | RNF2 | chr1:185056770-185056789 | GTCATCTTAGTCATTACCTG | - | FWD |
| Signal transducer and activator of transcription 3 | STAT3 | chr17:40481574-40481594 | CTCTGCAGAATTCAAACACT | - | REV |
| Elastase, neutrophil expressed | ELANE | chr19:853291-853310 | GAGCCCATAACCTCTCGCGG | + | FWD |
| FANCF FA complementation group F | FANCFA | chr11:2264733522647354 | GGAATCCCTTCTGCAGCACC | - | REV |
