# Supplementary material for: Rapid genome editing by CRISPR-Cas9-POLD3 fusion
Source: eLife. 2021 Dec 13;10:e75415. doi: 10.7554/eLife.75415 (PMC8747517; doi:10.7554/eLife.75415)
Supplement: Supplementary file 6. [file elife-75415-supp6.docx]

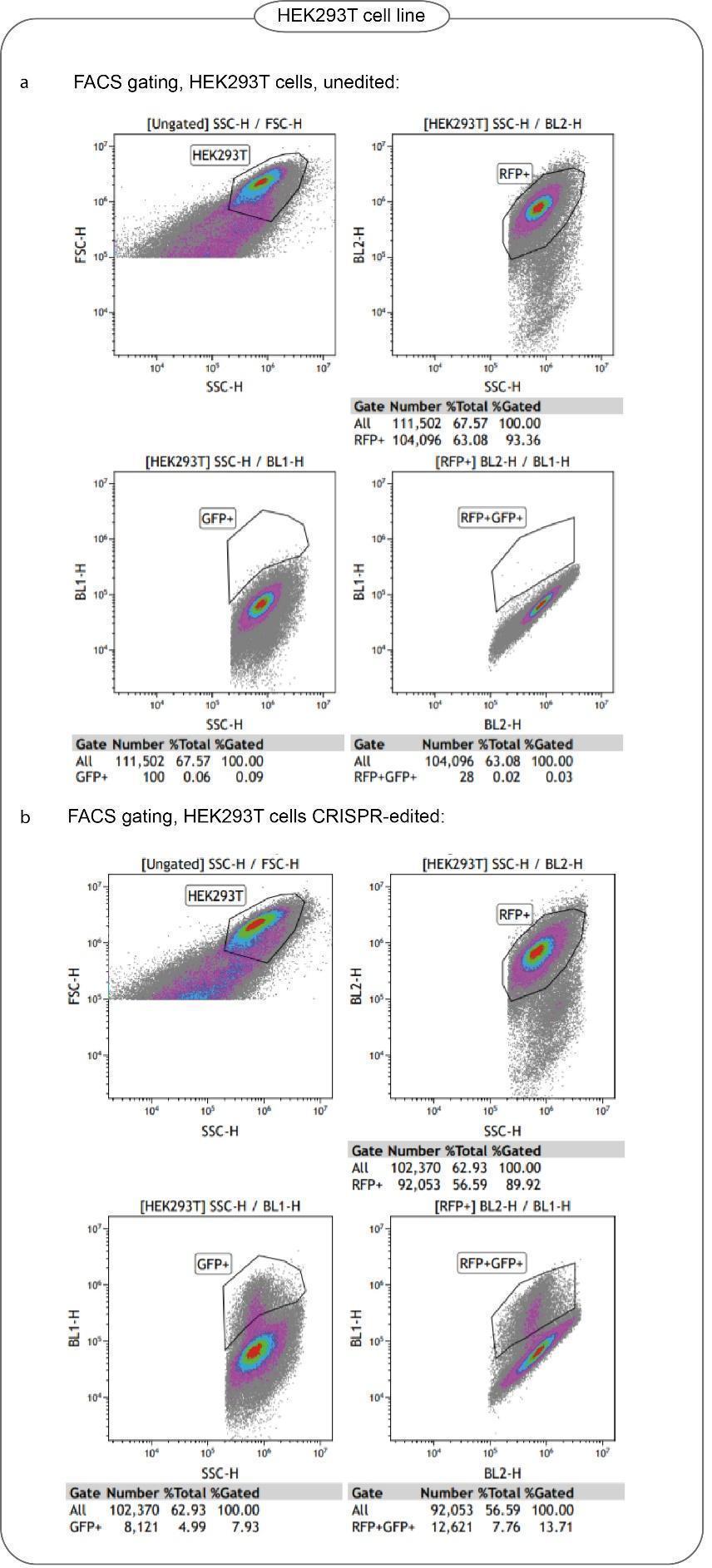


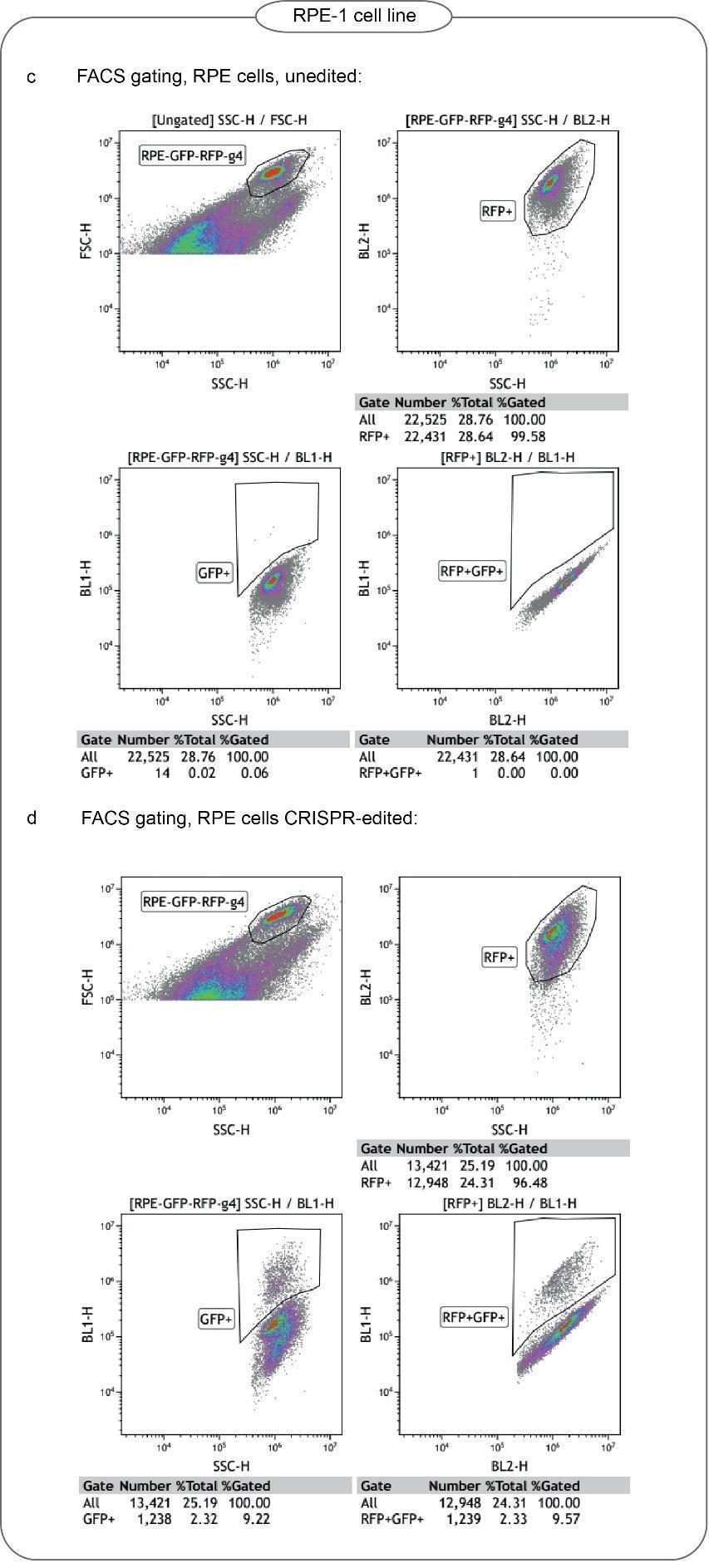


**
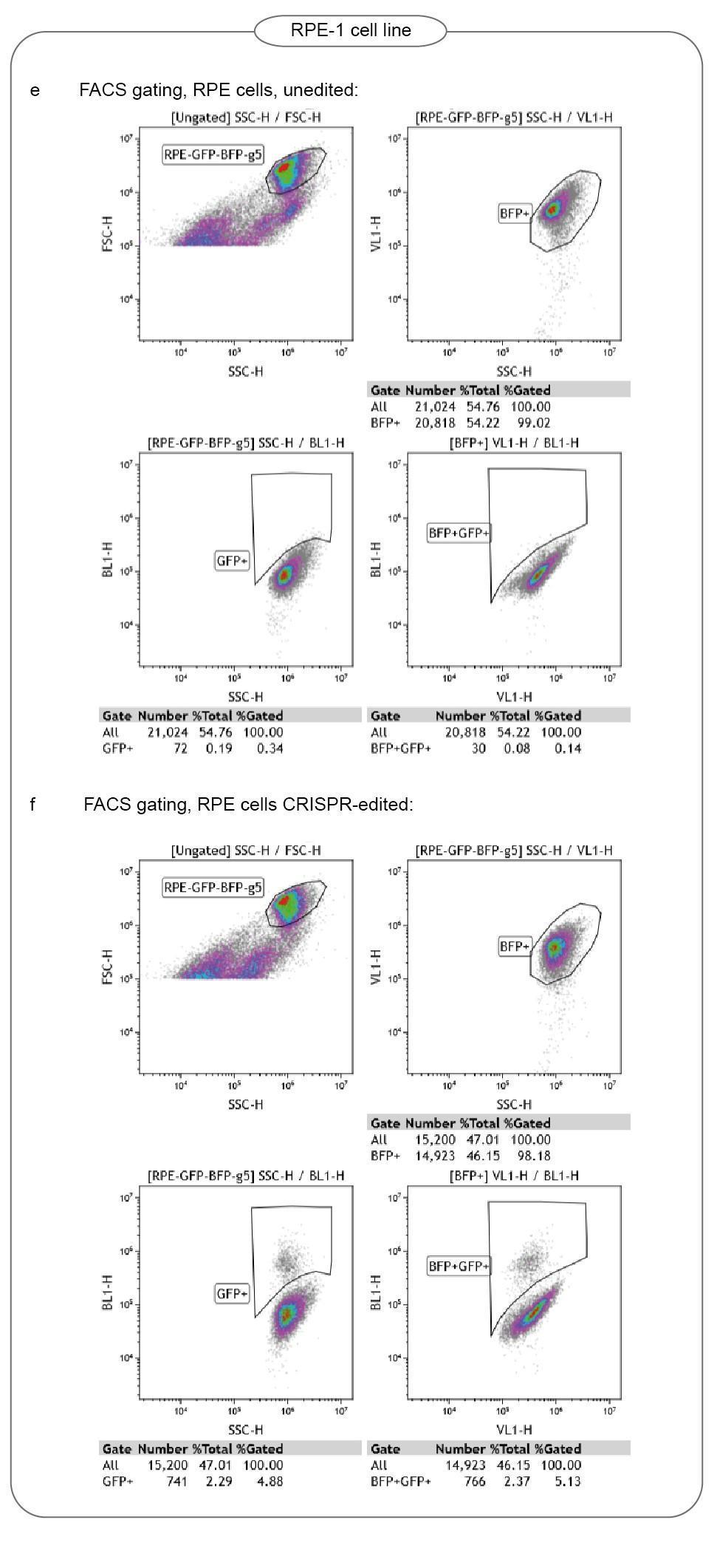
**

**FACS gating strategy**
**a-b.** HEK293T reporter cell line carrying mGFP-RFP color cassette. **c-d.** RPE-1 reporter cell line carrying mGFP-RFP color cassette. **e-f.** RPE-1 reporter cell line carrying mGFP-BFP color cassette.
