# Supplementary material for: Rapid genome editing by CRISPR-Cas9-POLD3 fusion
Source: eLife. 2021 Dec 13;10:e75415. doi: 10.7554/eLife.75415 (PMC8747517; doi:10.7554/eLife.75415)
Supplement: Supplementary file 7. [file elife-75415-supp7.docx]

**Supplementary file 7:**Sequences of ssDNA repair oligos, ddPCR primers and probes:

| **Target gene** | **DNA repair sequence** | **ddPCR primer fwd** | **ddPCR primer rev** | **ddPCR probe NHEJ** | **ddPCR probe HDR** | **ddPCR probe reference** |
| --- | --- | --- | --- | --- | --- | --- |
| **Enh 4-1** | ATGTGCTAGACAATATATTAGGTGTGGGATACAGTGAAATCAAATCCTTGagagtcaGAAGTTGACATTCTACTGACAGAAGACATTCAAGCAATACATG | TGTTGAGTGTGCCTGGAT | ACCCTTGCCTCTTTCCCT | CTTGCCCTCATGAAGTTGACATTC | CCTTGagagtcaGAAGTTGACATTCTA | TGGAGGACCGATCATGTGCTAGACA |
| **CTCF-1** | TTTGCTGCAAAGCGTCTTTCCCTCCGCCCCCTCTCTGGGCAGCACttatcaaCAGGTTTGCGAAAGTAAAGTAAGTGTGCCCTCTACTGGCAGCAGAGAT | AGGTGGCTGGAAACTTGT | GGAGCAACCAATCGCTATG | CAAACCTGAACGCGGGTGC | CGCAAACCTGTTGATAAGTGCTGC | CCTCGGACGCTCCTGCTCCT |
| **RNF2** | TAAGCAAAACATGGGAACTCAGTTTATATGAGTTACAACGAACACCTagttcggctcCTAAGATGACTGCCAAGGGGCATATGAGACGTGTAAACTGGGA | CTTCTTTATTTCCAGCAATGTCTC | GCCAACATACAGAAGTCAGG | ACCTCAGGTAATGACTAAGATGACTGC | CTAGTTCGGCTCCTAAGATGACTGC | CTGTGCAGACAAACGGAACTCAA |
| **STAT3** | CCGGCAGCCAGAGGCCCTTTGTGAAGGGGAGCTCCTCCCACATAtaAAcaGTTTGAATTCTGCAatagttCTGCCGTTGTTGGATTCTTCCATGTTCATC | GCTCCCTCAGGGTCTGTA | CCTGTGATTCAGATCCCG | CAAGTGTTTGAATTCTGCAGAGAGGC | TGAATTCTGCAatagttCTGCCGTT | AAAGGCAGGTGTCCTGTGA |
| **ELANE** | GTTTTCGAAGATGCGCTGCACGGCGAACACCTGCCGGGTGGGCTCttGatGtGAGAGGTTATGGGCTCCCAGGACCACCCGCACCGCGCGGACGTTTCTG | GGATGGGGACGACAAGG | GCACCTGGAGAATCACGA | CTCGCGGCGGGAGC | CATAACCTCTCaCatCaaGAGCCC | ACCCCGTAAACTTGCTCAACGA |
| **FANCF** | GGTGCTGACGTAGGTAGTGCTTGAGACCGCCAGAAGCTCGGAAAAGCGATtgtatcGCTGCAGAAGGGATTCCATGAGGTGCGCGAAGGCCCTACTTCCG | ATGGATGTGGCGCAGGTA | CGCGGATGTTCCAATCAG | CGATCCAGGTGCTGCAGAA | AGCGATtgtatcGCTGCAGAA | TCACCTTGGAGACGGCGAC |
| **GFP** | GAACGGCCACAAGTTCAGCGTGTCCGGCGAGGGCGAAGGCGACGCCACATATGGCAAGCTGACCCTGAAGTTCATCTGCACCACCGGCAAGCTGCCCGTG | ATCCTGGTGGAACTGGATG | GAAGTCGTGCTGCTTCATAT | GCCGCTGCCAAGCTGAC | CGCCACATATGGCAAGCTGAC | GCGACGTGAACGGCCAC |

**Letters written in non-capital font in the “DNA repair sequence” column represent nucleotides that are different from the original WT gene sequence.*

**
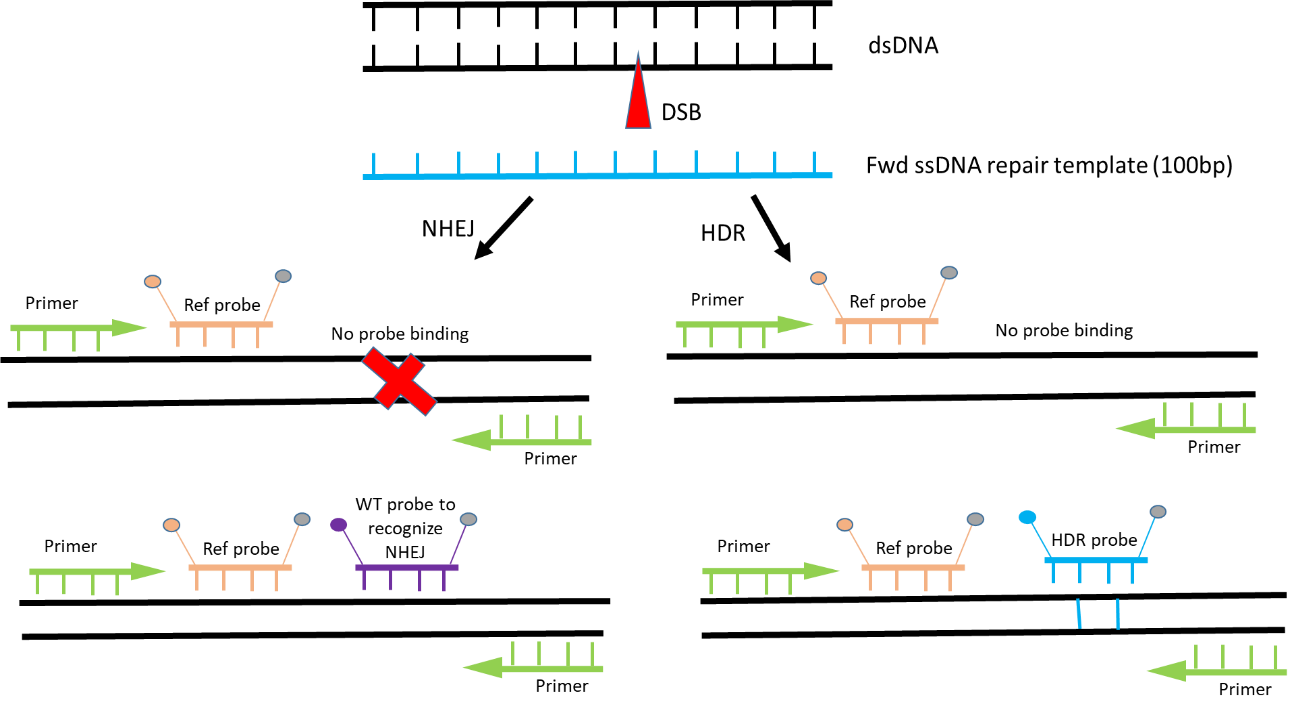
**
**Supplementary file 7 – figure 1. Quantifying CRISPR editing by droplet digital PCR (ddPCR).**

Primers flank the DNA sequence that surrounds the CRISPR-Cas9 target site. Reference probe binds to the unedited sequence and detects the presence of the amplicon in the droplet. NHEJ probe binds to the WT sequence at the Cas9 target site and drops off if the site mutates because of editing. HDR probe binds to the knock-in DNA sequence from the repair DNA template.

**
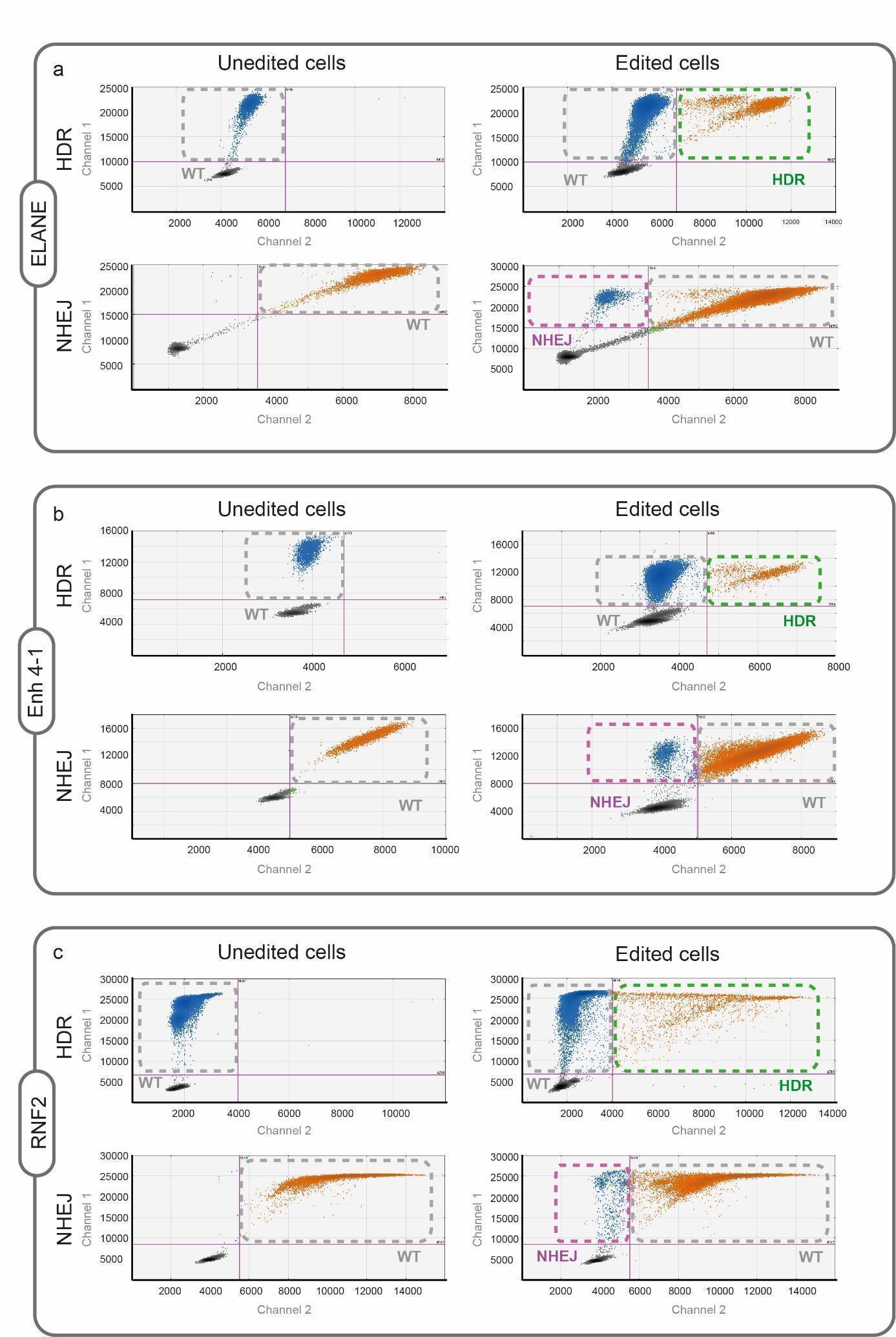
**

**
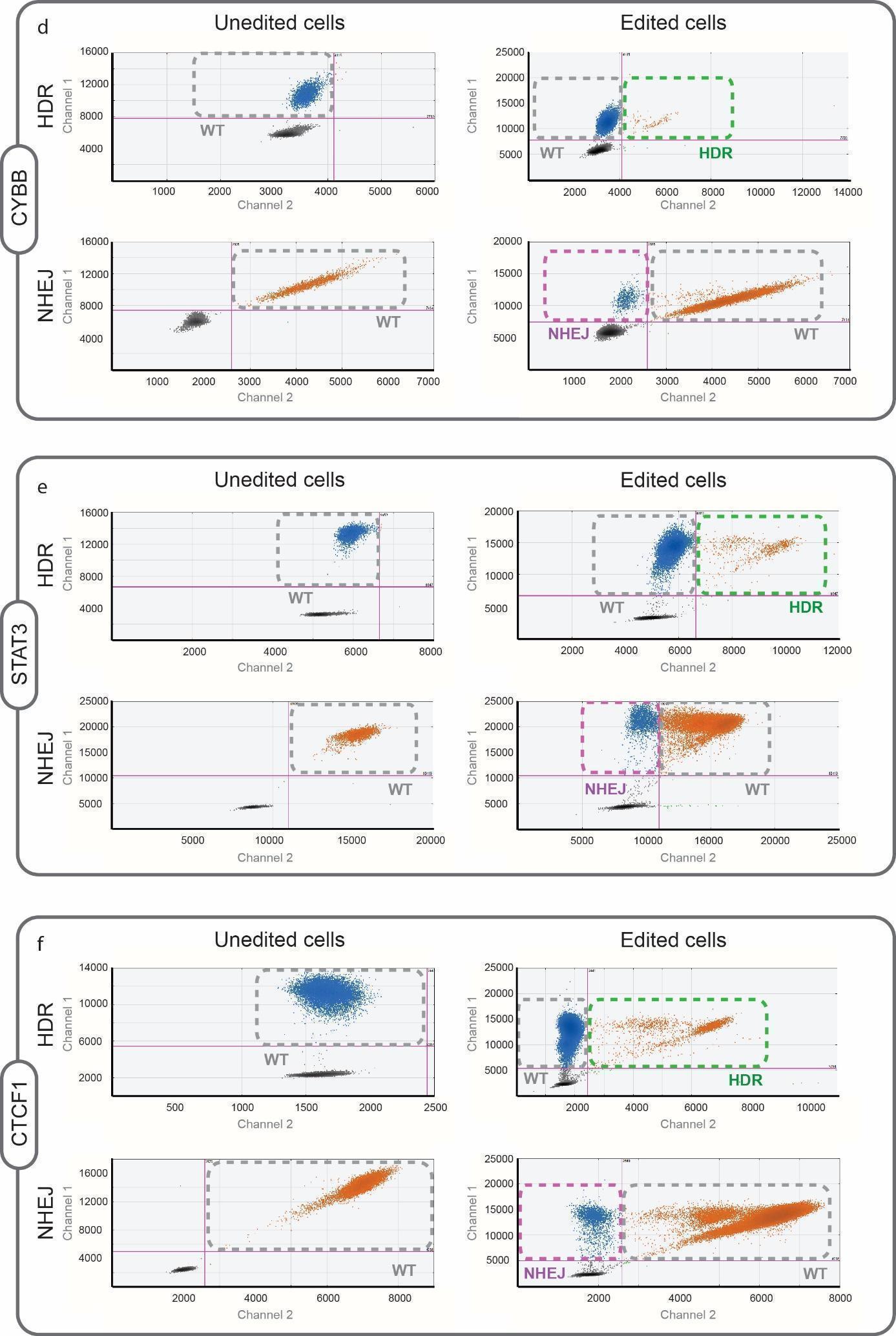
**

**
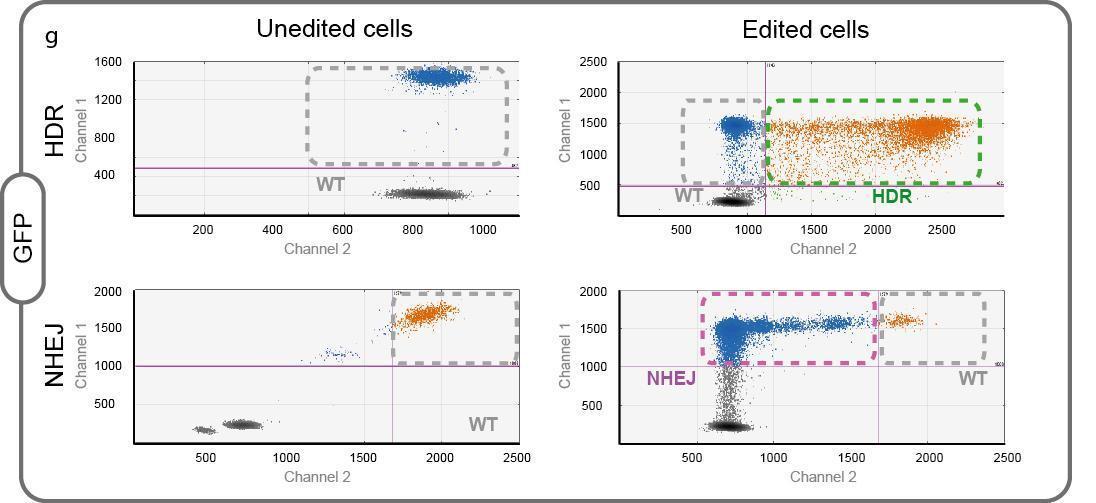

Supplementary file 7 – figure 2. Examples of the ddPCR gating for quantifying HDR and NHEJ.**

For each set, the droplet distribution in the negative control is shown on the left and the CRISPR-edited samples on the right.
